# 21.1 STRESS AND COGNITION IN YOUTH AT CLINICAL RISK FOR PSYCHOSIS

**DOI:** 10.1093/schbul/sby014.085

**Published:** 2018-04-01

**Authors:** Elaine Walker

**Affiliations:** Emory University

## Abstract

**Background:**

Heightened Stress has been shown to have acute adverse effects on cognitive function, and both stress and cognitive deficits have been found to be inversely associated with hippocampal volume reduction in both healthy and clinical samples. Further, heightened stress and cognitive deficits have been observed in individuals at clinical high risk (CHR) for psychosis in the North American Prodrome Longitudinal Study (NAPLS-2) as well as other studies of CHR groups. The present study utilizes data from NAPLS-2 to examine the relation of acute stress and hippocampal volume with cognitive performance in healthy youth and those at CHR for psychosis. Both the independent and additive relations of daily stress and hippocampal volume (HV) on cognition are examined.

**Methods:**

The sample was 666 participants (CHR=476; HC=190) who completed MRI scans, as well as measures of stress and cognitive function at the NAPLS-2 baseline assessment. The self-report stress measure was the Daily Stress Inventory (DSI) and cognitive performance was assessed with tests from the Measurement and Treatment Research to Improve Cognition in Schizophrenia (MATRICS) battery (the Brief Assessment of Cognition in Schizophrenia [BACS]: Symbol Coding, Category Fluency: Animal Naming Trail Making Test: Part A [TMT], Continuous Performance Test—Identical Pairs [CPT-IP], Hopkins Verbal Learning Test—Revised [HVLT-R], Brief Visuospatial Memory Test—Revised [BVMT-R], Neuropsychological Assessment Battery® (NAB): Mazes, and Wechsler memory Scale [WMS]). Hierarchical Linear regression analyses were conducted controlling for subject age, sex and intracranial volume (ICV) with test scores as the dependent measures and DSI scores and hippocampal volume as the predictors.

**Results:**

As expected, across subject groups, age was positively associated with performance on all of the cognitive measures, and females scored higher than males on the CPT-IP, WMS, and TMT. ICV was positively linked with performance on the TMT, WMS, NAB Mazes, CPT-IP, and BACS. For HC and CHR subjects combined, after entering covariates (sex, age and ICV), DSI, but not HV, was inversely associated with performance on the BVMT, HVLT, CPT-IP, and WMS. Neither DSI nor HV predicted performance on the NAB mazes or the BACS, beyond the variance accounted for by covariates. When analyses were conducted separately for the HC and CHR groups, DSI was not predictive of performance.

**Discussion:**

The present findings indicate that self-reported daily stress is inversely associated with cognitive performance on a variety of measures, and that this relation is not mediated by HV or any of the covariates in the combined sample of HC and CHR youth. Because the two groups differ in DSI scores, with CHR youth showing significantly higher stress scores, the combined samples represent a broader range of scores than either group alone. Thus, the within-group range of DSI scores is constrained and DSI is not predictive of performance within group. Instead, it appears that elevated stress is one factor contributing to cognitive deficits in CHR youth.

